# Exploring the Associations of Inflammatory and Oxidative Stress Biomarkers with Pancreatic Diseases: An Observational and Mendelian Randomisation Study

**DOI:** 10.3390/jcm13082247

**Published:** 2024-04-12

**Authors:** Laura Vilà-Quintana, Esther Fort, Laura Pardo, Maria T. Albiol-Quer, Maria Rosa Ortiz, Montserrat Capdevila, Anna Feliu, Anna Bahí, Marc Llirós, Esther Aguilar, Adelaida García-Velasco, Mireia M. Ginestà, Berta Laquente, Débora Pozas, Aleix Lluansí, Ville Nikolai Pimenoff, Victor Moreno, Libadro Jesús Garcia-Gil, Eric J. Duell, Robert Carreras-Torres, Xavier Aldeguer

**Affiliations:** 1 Digestive Diseases and Microbiota Group, Department of Gastroenterology, Girona Biomedical Research Institute (IDIBGI), Hospital Universitari de Girona Dr. Josep Trueta, 17190 Salt, Spain; lvila@idibgi.org (L.V.-Q.); efort.girona.ics@gencat.cat (E.F.); lpardo.girona.ics@gencat.cat (L.P.); mcapdevila@idibgi.org (M.C.); afeliu@idibgi.org (A.F.); abahi@idibgi.org (A.B.); eaguilar@idibgi.org (E.A.); dpozas@idibgi.org (D.P.); jesus.garcia@udg.edu (L.J.G.-G.); 2 General and Digestive Surgery Group, Department of Surgery, Girona Biomedical Research Institute (IDIBGI), Hospital Universitari de Girona Dr. Josep Trueta, 17007 Girona, Spain; talbiol.girona.ics@gencat.cat; 3 Department of Pathology, Hospital Universitari de Girona Dr. Josep Trueta, 17007 Girona, Spain; rortizduran.girona.ics@gencat.cat; 4 Bioinformatics and Bioimaging (BI-SQUARED) Research Group, Biosciences Department, Faculty of Sciences, Technology and Engineerings, Universitat de Vic—Universitat Central de Catalunya, 08500 Vic, Spain; marc.lliros@uvic.cat; 5 Precision Oncology Group, Girona Biomedical Research Institute (IDIBGI), Institut Català d’Oncologia (ICO), Hospital Universitari de Girona Dr. Josep Trueta, 17007 Girona, Spain; agvelasco@iconcologia.net; 6 Hereditary Cancer Program, Oncobell Program, CIBERONC, Institut Català d’Oncologia (ICO), Institut d’Investigació Biomèdica de Bellvitge (IDIBELL), 08908 Barcelona, Spain; mmorell@iconcologia.net; 7 Medical Oncology Department, Institut Català d’Oncologia (ICO), Institut d’Investigació Biomèdica de Bellvitge (IDIBELL), 08908 Barcelona, Spain; blaquente@iconcologia.net; 8 Institut de Recerca Sant Joan de Déu (IRSJD), Hospital Sant Joan de Déu, 08950 Barcelona, Spain; aleix.lluansi@sjd.es; 9 Department of Clinical Science, Intervention and Technology—CLINTEC, Karolinska Institutet, 141 52 Huddinge, Sweden; ville.pimenoff@ki.se; 10 Unit of Population Health, Faculty of Medicine, University of Oulu, 90570 Oulu, Finland; 11 Catalan Institute of Oncology (ICO), Institut de Recerca Biomedica de Bellvitge (IDIBELL), 08908 Barcelona, Spain; v.moreno@iconcologia.net; 12 Department of Clinical Sciences, Faculty of Medicine and health Sciences and Universitat de Barcelona Institute of Complex Systems (UBICS), University of Barcelona (UB), L’Hospitalet de Llobregat, 08028 Barcelona, Spain; 13 Consortium for Biomedical Research in Epidemiology and Public Health (CIBERESP), 28029 Madrid, Spain; 14 Cancer Epidemiology Research Program, Unit of Nutrition and Cancer, Institut Català d’Oncologia (ICO), Institut d’Investigació Biomèdica de Bellvitge (IDIBELL), 08908 Barcelona, Spain; eduell@idibell.onmicrosoft.com

**Keywords:** pancreatic ductal adenocarcinoma, chronic pancreatitis, biomarkers, oxidative stress, Mendelian randomisation analysis

## Abstract

Identifying biomarkers linked to pancreatic ductal adenocarcinoma (PDAC) and chronic pancreatitis (CP) is crucial for early detection, treatment, and prevention. **Methods:** Association analyses of 10 serological biomarkers involved in cell signalling (IFN-γ, IL-6, IL-8, IL-10), oxidative stress (superoxide dismutase (SOD) and glutathione peroxidase (GPx) enzyme activities, total glutathione (GSH), malondialdehyde (MDA) levels), and intestinal permeability proteins (zonulin, I-FABP2) were conducted across PDAC (*n* = 12), CP (*n* = 21) and control subjects (*n* = 23). A Mendelian randomisation (MR) approach was used to assess causality of the identified significant associations in two large genetic cohorts (FinnGen and UK Biobank). **Results:** Observational results showed a downregulation of SOD and GPx antioxidant enzyme activities in PDAC and CP patients, respectively, and higher MDA levels in CP patients. Logistic regression models revealed significant associations between CP and SOD activity (OR = 0.21, 95% CI [0.05, 0.89], per SD), GPx activity (OR = 0.28, 95% CI [0.10, 0.79], per SD), and MDA levels (OR = 2.05, 95% CI [1.36, 3.08], per SD). MR analyses, however, did not support causality. **Conclusions:** These findings would not support oxidative stress-related biomarkers as potential targets for pancreatic diseases prevention. Yet, further research is encouraged to assess their viability as non-invasive tools for early diagnosis, particularly in pre-diagnostic CP populations.

## 1. Introduction

Pancreatitis and pancreatic cancer are significant diseases with a considerable medical impact. Pancreatic ductal adenocarcinoma (PDAC) is the predominant form of pancreatic cancer in Western countries, with a dire prognosis when detected late [1,2,3]. Late presentation, lack of effective screening, complex biology, and limited treatment options contribute to poor outcomes [1,4]. Pancreatitis, marked by inflammation and scarring of the pancreas, leads to irreversible loss of pancreatic function [5]. Early diagnosis is challenging due to nonspecific symptoms [6]. Chronic pancreatitis (CP) is a known risk factor for developing PDAC, and the risk increases with the duration of the disease [7,8,9]. PDAC and CP patients etiologically share basal tissue inflammation and several modifiable risk factors, where alcohol intake has the strongest effect, as most CP cases are alcohol related. Alcohol has multiple effects on pancreatic function and strongly affects the risk of smoking and other risk factors [3,5]. Currently, carbohydrate antigen 19-9 (CA19-9) is the sole approved biomarker for PDAC diagnosis and monitoring [10]. Nonetheless, CA19-9 has limitations in terms of specificity and sensitivity [11,12,13]. Therefore, identifying sensitive, specific, and cost-effective biomarkers associated with pancreatic diseases (PDAC and CP) for primary prevention and/or early detection is still an unresolved problem [10]. The contributions of immune cells to the pathogenesis of both PDAC and CP are receiving increased interest [14]. Alterations in cytokine levels, particularly IL-1β, IL-6, IL-8, IL-10, transforming growth factor (TGF), and vascular endothelial growth factor (VEGF), which have either pro-inflammatory or immunosuppressive effects, have been described as potential biomarkers, as they promote a favourable environment for the development and progression of pancreatic diseases [13]. Combining measurements of pro-inflammatory (i.e., IFN-γ, IL-6, IL-8) and immunosuppressive cytokine levels (i.e., IL-10) can help elucidate the dysregulation in the balance between both types of cytokines, which is typically exhibited in chronic inflammatory conditions. Also, combining measurements of both type of cytokines may yield predictive biomarkers for disease outcomes or treatment responses. 

Similarly, reactive oxygen and nitrogen species (RONS), which are stimulated by risk factors, provoke local and systemic oxidative stress and inflammation in pancreatic diseases [15]. Oxidative stress biomarkers, such as MDA and GSH, have been observed to be elevated in several types of cancer, including PDAC [16]. Antioxidant enzymes are essential for protecting cells against RONS and can also be used as biomarkers. Several studies have been conducted on the activity of antioxidant enzymes such as superoxide dismutase (SOD), catalase (CAT), and glutathione peroxidase (GPx) in CP and PDAC patients, and the findings revealed that the antioxidant capacity was lower in these patients than in controls [15,16]. Thus, serological measurements of GSH, the main ROS scavenger molecule, MDA, one of the several byproducts of lipid peroxidation processes which occurs with prolonged exposure to oxygen radicals, and GPx and SOD activity levels, two of the main antioxidants to reduce/neutralise ROS, may all provide a more comprehensive understanding of the imbalance between the production of ROS and the ability of cells to detoxify them, which is associated with the diseases and contributes to tissue damage, inflammation, and cancer progression. 

Additionally, gut barrier homeostasis regulates intestinal permeability and systemic inflammation; thus, biomarkers related to gut barrier homeostasis play an important role in the pathogenesis of pancreatic diseases [17,18]. Current methods for the evaluation of intestinal permeability involve invasive procedures and are time-consuming; therefore, several endogenous plasma-circulating proteins, such as intestinal fatty acid-binding protein (I-FABP) and zonulin, have been proposed as non-invasive biomarkers for the measurement of intestinal permeability [19]. For that reason, the measurement of plasma-circulating I-FABP and zonulin levels may offer insights into gut barrier dysfunction and its potential contribution to the pathophysiology of PDAC and CP. However, further studies need to evaluate whether these markers are causal factors for pancreatic diseases, which could increase their usefulness as biomarkers for primary prevention and early screening. Randomised controlled trials are the gold standard for establishing causation; however, they are unfeasible for most of these target biomarkers. In this scenario, Mendelian randomisation (MR) is a method that provides evidence of causality by utilizing human germline genetic variation as an instrument for identifying a relevant biomarker. As germline genetic variation is randomly inherited and fixed at conception, the results of MR analyses should be largely independent of both confounding and reverse causation and should be able to discern between correlation and causation [20]. 

The present study aims to analyse the associations between ten serological biomarkers involved in cell signalling, oxidative stress, and intestinal permeability and pancreatic diseases (PDAC and CP) and assess the causality of the identified significant associations. 

## 2. Materials and Methods

Fifty-six (56) participants were recruited at the Hospital Universitari Dr. Josep Trueta (HUJT, Girona, Spain) between September 2020 and June 2022. Patients were divided into 2 case groups, PDAC (*n* = 12) and CP (*n* = 21), and there was one control group (*n* = 23). The diagnosis of PDAC and CP depended on blood tests, specifically the measurement of CA19-9 levels, in addition to the evaluation of clinical features through computed tomography (CT) and/or magnetic resonance imaging (MRI), as well as biopsy or fine-needle aspiration using endoscopic ultrasound (EUS-FNA). The disease progression status of the PDAC patients was as follows: 9 patients had stage II disease (4 patients had stage IIA disease, 5 patients had stage IIB disease), 2 patients had stage I disease (1 patient had stage IA, and the other had stage IB), and 1 patient had stage IIIA disease. Three out of the twelve PDAC patients (two considered at stage IIA and one considered at stage IIB) received neoadjuvant therapy prior to surgery, which may have affected the determination of disease stage. Alcoholic CP was diagnosed in 18 patients, obstructive CP in 1 patient, and idiopathic CP in 2 patients. Exocrine dysfunction (faecal elastase <200 μg/g) was found in 16 CP patients. No gastrointestinal complications were reported in the CP patients enrolled in the study. Healthy controls were defined as individuals with gastric reflux, those being monitored for benign digestive diseases, or those with negative results from population screening for colorectal cancer. The exclusion criteria for all three groups were as follows: decompensated diabetes mellitus, concomitant malignancies; immunosuppressive therapy including chemotherapy, acute pancreatitis or acute relapse of CP, antibiotic or laxative treatment within one month prior to the study, antioxidant and/or anti-inflammatory treatment at the time of inclusion, pregnancy or breastfeeding, and disability to give informed consent. Demographic information, clinical features, and medication intake were documented at study entry. Tobacco smoking status (current, former, never) was determined by cigarette intake upon enrolment, while alcohol consumption (high, moderate, low) was categorised by weekly standard drink quantity (≥7, 2–7, <2, respectively). Blood samples were taken after an overnight fast and collected in vacutainer tubes with ethylenediaminetetraacetic acid (EDTA) to avoid coagulation, and plasma was prepared by centrifugation at 3000 rpm at 4 °C for 10 min.

A total of 10 plasma biomarkers were assessed using enzyme-linked immunosorbent assay (ELISA) kits following the manufacturer’s protocols. For cytokine level analysis, a panel of 4 different cytokines (IL-6, IL-8, IL-10, and interferon γ (IFN-γ)) from the MILLIPLEX^®^ human cytokine/chemokine/growth factor panel A immunology multiplex assay was used (Merck Life Science SLU, Darmstadt, Germany, Catalogue #HCYTA-60K). For the analyses of oxidative stress parameters (SOD and GPx enzyme activities plus lipid peroxidation and glutathione), the glutathione peroxidase assay kit, glutathione assay kit (Cayman Chemical, Ann Arbor, Michigan, USA, with catalogues #703102 and #703002, respectively), lipid peroxidation (MDA) assay kit, and superoxide dismutase (SOD) activity assay kit (Sigma-Aldrich-Merck SA, Darmstadt, Germany, with catalogues #MAK085 and #CS0009, respectively) were used. Finally, for the analyses of the two intestinal permeability proteins (zonulin and I-FABP), we used the human zonulin ELISA kit (Elabscience^®^, Houston, TX, USA, catalogue #E-EL-H5560) and the human FABP2/I-FABP ELISA kit PicoKine^®^ (Boster Biological Technology, Pleasanton, CA, USA, catalogue #EK1410).

All the statistical analyses were performed using R software (R 4.2.3, https://www.r-project.org/; accessed in March 2023). A statistically significant difference in categorical demographic data among groups was detected using Pearson’s chi-squared test. Values at 5 standard deviations (SDs) from the mean were considered outliers and further removed from the dataset. The data were normally distributed and were tested with the nonparametric Kolmogorov-Smirnov test. Differences among groups were tested with one-way analysis of variance (ANOVA). Linear regression models were used to evaluate associations between standardised demographic parameters and study biomarkers. Logistic regression models were used to evaluate the associations of study biomarkers with pancreatic disease outcome (PDAC or CP). Regressions were adjusted for sex, age, and body mass index (BMI). Logistic ridge regression (L2 regularisation) was used when multicollinearity occurred. L2 regularisation adds a regularisation term to the model estimates, penalizing large coefficients and preventing overfitting [21]. The logistic ridge function of the ridge package in R was used to fit those models, and the ridge regression parameter (lambda) was chosen automatically using the method proposed by Cule et al. (2013) [22]. The odds ratio (OR) with its corresponding 95% confidence interval (95% CI) was calculated, considering *p* values < 0.005 statistically significant after adjusting for multiple testing by Bonferroni and <0.05 suggestively significant. We performed an estimation of the minimum effect size to be detected with enough power (1 − β = 0.8) considering a significance level of 0.05 (α = 0.05) and assuming a standard deviation of the outcome in the population of 1 (σ = 1). For the PDAC group, the study has enough statistical power for odds ratio over 2.71 and lower than 0.37, while for the CP group over 2.33 and lower than 0.43 [23,24]. 

A two-sample MR was used to examine the associations between selected biomarkers and pancreatic disease risk. In the two-sample MR, instrument–exposure and instrument–outcome associations are obtained from different study sources and combined as a ratio to estimate the effects of the exposures on the outcomes [25]. Genetic instruments used were single nucleotide polymorphisms (SNPs) found to be associated with independent European studies, with *p* values < 0.01 for target studies (i.e., SOD and GPx activity) [26,27] and with *p* values < 1 × 10^−7^ for genome-wide association studies (GWASs) (i.e., MDA levels) [28]. When association parameters (effect size and standard error (SE)) of genetic instruments with biomarkers were not reported, they were estimated assuming a standardised trait with a mean of 0 and a SD of 1, as suggested by Zhu et al., 2016 [29]. The strength of associations between the genetic instrument and tested biomarkers was reflected in the F-statistic, which is inversely related to weak instrument bias, with 10 being the minimum estimation for an F-statistic to avoid a bias of this nature. The F-statistic was estimated as a function of the explained phenotypic variance (R^2^), the sample size, and the number of genetic variants [30]. The explained phenotypic variance (R^2^) for an SNP was estimated as a function of the effect size for the risk factor in SD units and the minor allele frequency [31]. The strength of the MDA instrument could not be calculated, because the association parameters of the genome-wide study reflected units of increase in residuals from a regression analysis on a non-standardised measure.

Pancreatic disease data were obtained from two large and independent European ancestry biobank cohorts, the UK Biobank [32] and FinnGen [33] studies. The UK Biobank data were obtained from the GWAS Catalogue (GCST90041814 and GCST90044205; accessed in August 2023), comprising 587 cases and 455,761 controls for PDAC and 322 cases and 456,026 controls for CP. The FinnGen study included the DF9 data release (https://r9.finngen.fi/; accessed in August 2023) and included 692 patients and 287,137 controls for PDAC and 3320 patients and 330,903 controls for CP. When association parameters for instrumental SNPs were not identified in the pancreatic disease GWAS, LDlink (https://ldlink.nih.gov/; accessed in August 2023) was used to identify proxy SNPs (R^2^ for linkage disequilibrium > 0.8). The TwoSampleMR package (https://mrcieu.github.io/TwoSampleMR/index.html; accessed in September 2023) in R was used to examine the causal relationship between the biomarkers and PDAC or PC. The Wald ratio method was used for the primary analysis when only one SNP was associated with each biomarker. If more than one SNP was associated, the inverse-variance weighted (IVW) method was used [34]. No other sensitivity analyses were performed because of the low number of SNPs identified for each biomarker.

## 3. Results

### 3.1. Baseline Characteristics

A total of 56 patients were enrolled in the present study and was comprised of 12 patients with PDAC, 21 patients with CP, and 23 HCs. Forty-four percent of patients were female, and fifty-six percent were male, with males being more common in the CP group and females being more common in the PDAC group (*p* value = 0.004). Cancer patients were older than were those in the other groups (*p* value = 0.005) (Table 1). Fifty-nine percent of patients were current smokers or former smokers, and forty percent had high or moderate weekly alcohol intake; both behaviours were more prevalent among CP patients (*p* value = 0.0005 for both smoking and drinking status) (Table 1). Finally, among the other tested clinical parameters, type II diabetes was unevenly distributed among the groups (8.7% of HCs, 33.3% of patients with PDAC, and 61.9% of patients with CP; *p* value = 0.002). However, insulin treatment was similarly distributed among the groups (50.0% of HCs, 100.0% of patients with PDAC, and 69.2% of patients with CP; *p* value = 0.77) (Table 1).

### 3.2. Association Analysis of Serological Biomarkers

**Figure 1:**
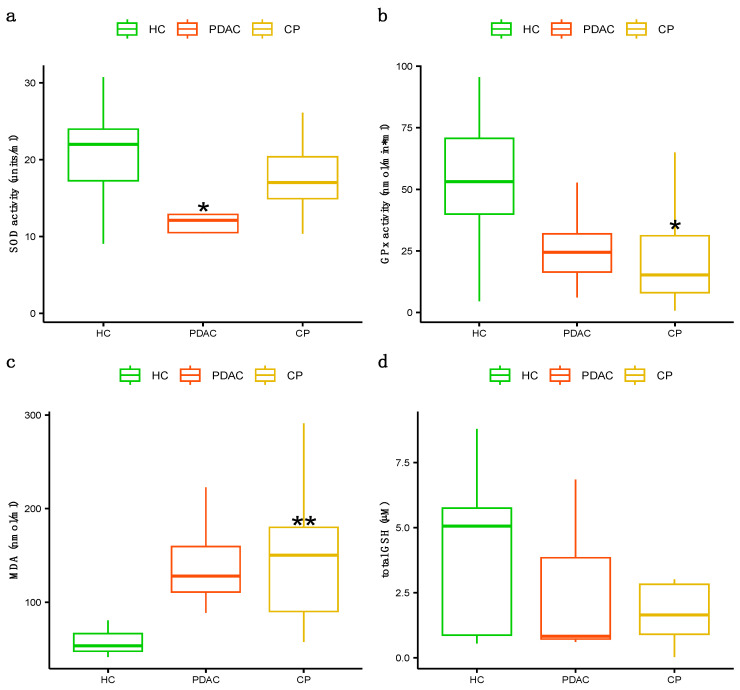
Plasma concentration of antioxidant enzymes activity plus oxidative stress markers: (**a**) plasma concentration of SOD enzyme activity, (**b**) plasma concentration of GPx enzyme activity, (**c**) plasma concentration of MDA levels, (**d**) total plasma concentration of glutathione (GSH). As for parametric variables, data are expressed as mean ± SD. PDAC, CP vs. control: ** *p* < 0.005, * *p* < 0.05. (One-way analysis of variance (ANOVA) with Tukey HSD post hoc test). HCs (healthy controls), PDAC (pancreatic cancer), CP (chronic pancreatitis).

**Figure 2:**
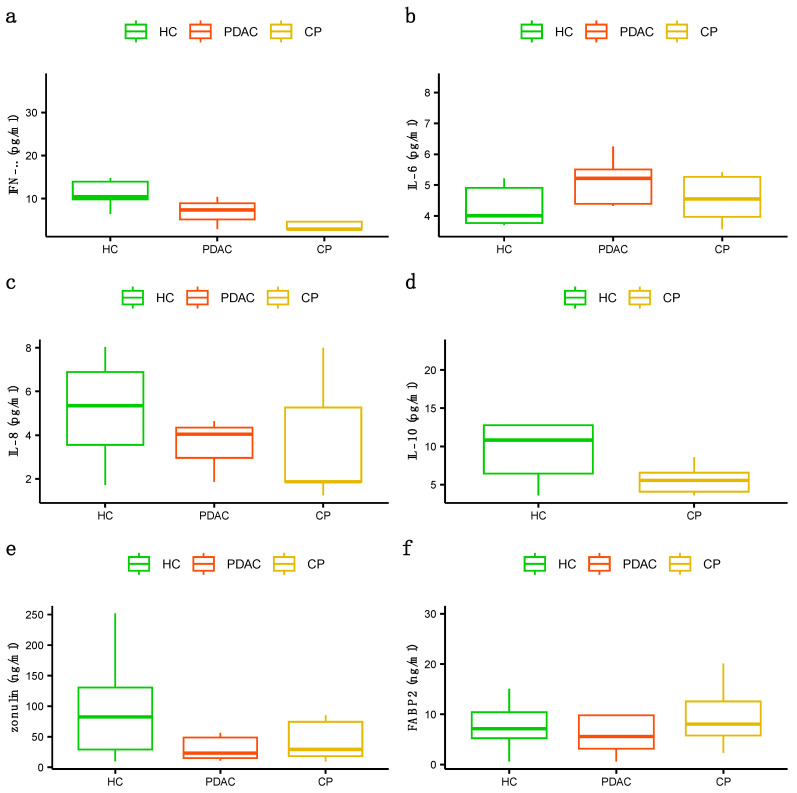
Plasma levels of studied cytokines and intestinal permeability proteins: (**a**) plasma levels of IFN-γ, (**b**) plasma levels of IL-6, (**c**) plasma levels of IL-8, (**d**) plasma levels of IL-10, (**e**) plasma levels of zonulin, (**f**) plasma levels of intestinal FABP. As for parametric variables, data are expressed as mean ± SD. (One-way analysis of variance (ANOVA) with Tukey HSD post hoc test). HCs (healthy controls), PDAC (pancreatic cancer), CP (chronic pancreatitis).

Decreased plasma antioxidant activity of SOD was found in the PDAC patients compared to the controls (*p* value = 0.006), while the antioxidant activity of the enzyme GPx was found to be lower in the patients with CP than in the controls (*p* value = 0.006) (Figure 1a,b). Moreover, higher levels of the oxidative stress marker MDA were found in patients with CP than in controls (*p* value = 0.0005), but no differences were found in the total GSH plasma concentration between the groups (Figure 1c,d). Finally, we did not find statistically significant differences in IFN-γ, IL-6, IL-8, or IL-10 cytokine levels or intestinal permeability protein levels of zonulin and FABP2 (Figure 2).

Correlations between biomarkers and demographic variables in control subjects were assessed to test for covariate parameters via regression models. As expected, we found correlations between biomarkers and sex, age, and BMI (sex vs. IL-8, *p* value = 0.002; age vs. SOD activity, *p* value = 0.01; BMI vs. IL-10, *p* value = 0.05). Therefore, we constructed logistic regression models controlling for sex, age, and BMI (Table 2). A one-SD increase in GPx activity was associated with a lower risk of CP (OR 0.28 95% CI [0.10, 0.79]). Similarly, the results showed that the risk for CP decreased (OR 0.21; 95% CI [0.05, 0.89]) for a one-SD increase in SOD activity. The MDA data for the CP group were fitted using a logistic ridge regression model (L2 regularisation) due to the presence of multicollinearity. We found that a higher risk of CP was associated with an increase in the MDA concentration (OR 2.05; 95% CI [1.36, 3.08]). No significant associations were found for cytokine levels or intestinal permeability protein levels in the PDAC or CP groups. 

### 3.3. Mendelian Randomisation Analyses

Genetic instruments (SNPs) for SOD activity, GPx activity, and MDA levels were identified. The SNPs rs4880 and rs1050450 are common missense variants associated with SOD (Val16Ala in the SOD2 gene) and GPx (Pro200Leu in the GPX1 gene) enzyme activity, respectively, identified in target studies [26,27]. The SOD activity instrument showed strength for MR analysis (F-statistic = 20.95); however, the strength of the GPx activity instrument was modest (F-statistic = 6.8) (Table 3). In the case of MDA levels, the SNPs rs33965115, rs80018995, and rs59408048 were identified in a genome-wide setting [28]. MR analysis of the UK Biobank and FinnGen cohorts did not reveal significant causal relationships between the studied biomarkers and pancreatic diseases (Table 4).

## 4. Discussion

Initially, we observed that sex, age, and BMI affected the distribution of several biomarkers. Obesity has been described as a state of chronic low-grade inflammation and is one of the factors that contributes to oxidative stress in obese patients [35]. However, controversial results have been published regarding pro- and anti-inflammatory cytokine levels in obese subjects compared to healthy weight individuals. Schmidt et al., 2015 [36] reported that general obesity was associated with elevated serum levels of IL-10 and IFN-γ, confirming the upregulation of certain pro- and anti-inflammatory cytokines in individuals with obesity, while Charles et al., 2011 [37] reported that IL-10 was not associated with obesity. Other studies have suggested that obesity and metabolic syndrome increase oxidative stress and inflammatory markers, activating the inflammatory response through proinflammatory cytokines [38]. On the other hand, ageing is characterised by the progressive loss of tissue and organ function. The oxidative stress theory of ageing is based on the hypothesis that age-associated functional losses are due to the accumulation of RONS-induced damage [39]. The prevalence of oxidative stress is reported during aging, during which reduced plasma antioxidant potential is described [40]. Sex differences involving oxidative stress are common, as are sex differences in the stress response of cells and tissues; in females, cells are generally more resistant to heat- and oxidative stress-induced cell death [41].

The observational findings of our study were reduced plasma levels of the antioxidant SOD enzyme activity in PDAC patients and of the antioxidant GPx enzyme activity in CP patients, as well as increased levels of lipid peroxidation (MDA) in CP patients. Superoxide dismutase (SOD) converts superoxide (O^−2^) into hydrogen peroxide (H_2_O_2_), while peroxidase (GPx) converts H_2_O_2_ into water [42]. The first indication of an altered antioxidant profile in cancer versus normal cells was observed when the activity of the mitochondrial matrix form of the SOD enzyme was found to be decreased in many transformed versus normal cells [43]. Since then, numerous studies have investigated the role of oxidative stress in pancreatic diseases, although inconsistent results concerning the antioxidant defence system in CP and PDAC have been described [15,42,44,45,46,47]. Kodydkova et al., 2013 [15] reported that erythrocyte SOD activity was increased in PDAC patients compared to that in CP patients and controls. Fluctuations in the expression of SOD enzymes in cancer cells are still controversial [43,48]. Recent evidence has supported the conclusion that the enzyme is typically downregulated in the initial stages of tumour formation, but as cancer cells progress, the enzyme is upregulated [43]. The patients recruited for this study were not in advanced or metastatic stages of the disease, validating that SOD activity may be downregulated in the early stages of cancer development. The pathogenesis of CP indicates that ROS plays a critical role in activating the inflammatory cascade, recruiting inflammatory cells and causing tissue damage [45]. Studies of plasma and serum levels of antioxidant GPx activity and lipid peroxidation products indicate a generalised increase in oxidative conditions in CP [44,47,48], but inconsistent results concerning SOD activity in alcohol-related CP have been published [15]. Several reports have described decreased levels of SOD in pancreatic cells and serum compared to controls [42,47], while others have reported no differences in serum SOD activity [15,46]. These results support the hypothesis that antioxidant enzymes are downregulated and that lipid peroxidation is elevated in CP patients, with strong associations between these parameters and disease when demographic variables are controlled for. However, recent studies have indicated that low plasma levels of antioxidants do not necessarily indicate an increased oxidative state in the pancreas. Instead, decreased levels could be caused by malnutrition, maldigestion, or malabsorption, which are common among CP patients due to exocrine pancreatic insufficiency (EPI) and postprandial pain that discourages adequate food intake [44]. In our study, 16 out of 21 CP patients had exocrine pancreatic dysfunction. Thus, increased plasma levels of lipid peroxidation could be due to other sources of lipid peroxidation in addition to CP [44]. As stated above, the available literature regarding oxidative stress markers and antioxidant activities in inflammatory diseases and cancer is controversial, so their potential use as biomarkers for early diagnosis remains to be studied. 

In order to examine the causal role between the identified significant associations of oxidative stress-related biomarkers (i.e., SOD activity, GPx activity, and MDA levels) and CP risk, we performed MR analyses. None of the genetic instruments included in our study showed a significant causal role of biomarkers on CP. These results suggested that the observational results obtained could be a consequence of the disease; therefore, attempts to regulate antioxidant enzymes or lipid peroxidation will not be helpful for primary prevention of disease onset. However, the strength of the GPx activity instrument used was modest, which could have biased our results.

The main limitation of this study is the small cohort used to investigate the associations of serological biomarkers to CP and PDAC. Hence, results of the present study may be biased and possibly related to other findings reported, such as EPI, and not the disease state. Therefore, data would need to be validated and tested in larger cohorts, specifically populations with pre-diagnostic pancreatic disease, in order to validate the potential diagnostic value of the discussed markers. Furthermore, we encourage future studies to further test the advanced oxidation protein products and oxidised low-density lipoproteins, as they could provide a more comprehensive view of the potential of oxidative stress-related biomarkers in PDAC development. 

## 5. Conclusions

This study showed a correlation between lower antioxidant capacity and higher oxidative stress marker levels with CP. MR analyses, however, indicated that these correlations can be a consequence of the disease rather than the cause. Hence, we encourage further studies to test the role of oxidative stress-related serological biomarkers as possible non-invasive tools for the early diagnosis of pancreatic diseases in clinical practice, with special interest on CP cases, and particularly in pre-diagnostic disease populations. These results warrant further studies to validate the associations of cytokines and intestinal permeability proteins in pancreatic diseases.

## Figures and Tables

**Table 1:** Baseline characteristics of enrolled patients by groups.

	HCs (*n* = 23)	PDAC (*n* = 12)	CP (*n* = 21)	*p*-Value
Sex, % (F/M)	52.2/47.8	66.7/33.3	14.3/85.7	0.004 **
Age, yrs	62.52 ± 10.89	69.17 ± 9.51	57.71 ± 7.05	0.005 **
Tobacco consumption, % (Current/Former/Never)	8.7/30.4/60.9	16.7/41.7/41.6	47.6/33.3/19.1	0.0005 **
Alcohol consumption, % (High/Moderate/Low)	8.7/4.3/87.0	16.7/8.3/75.0	0.0/80.9/19.1	0.0005 **
BMI, kg/m^2^	26.59 ± 4.80	26.25 ± 6.60	24.96 ± 5.16	0.64
Diabetes, % (yes/no)	8.7/91.3	33.3/66.7	61.9/38.1	0.002 **
Insulin treatment, % (yes/no)	50/50	100/0	69.2/30.8	0.77
Familiar pancreatic disease, % (yes/no)	4.3/95.67	20/80	10.5/89.5	0.34
PPI treatment, % (yes/no)	21.7/78.3	33.3/66.7	57.1/42.9	0.05
Previous digestive surgery, % (yes/no)	26.1/73.9	25/75	14.3/85.7	0.63

HCs: healthy controls; PDAC: pancreatic cancer; CP: chronic pancreatitis. F: females; M: males; BMI: Body Mass Index; PPI: Proton pump inhibitors. Parametric variables are expressed as mean ± SD for numerical data and in % for categorical data. Pearson’s chi-squared test for categorical data and ANOVA test for numerical data. ** *p* < 0.005.

**Table 2:** Association analysis for each study parameter and study group.

	PDAC			CP		
	OR	95% CI	*p*-Value	OR	95% CI	*p*-Value
FABP2 (ng/mL)	15.87	[0.12, 2029.81]	0.26	1.93	[0.61, 6.05]	0.26
Zonulin (ng/mL)	0.17	[0.01, 5.31]	0.31	0.61	[0.20, 1.81]	0.37
total GSH	0.46	[0.07, 3.04]	0.42	0.40	[0.13, 1.26]	0.12
GPx (nmol/min × mL)	0.33	[0.07, 1.65]	0.18	0.28	[0.10, 0.79]	0.02 *
SOD (units/mL)	0.03	[0.0007, 1.53]	0.08	0.21	[0.05, 0.89]	0.03 *
MDA (nmol/mL)	12.88	[0.46, 353.57]	0.13	2.05	[1.36, 3.08]	0.0005 **
IFN-γ (pg/mL)	0.02	[0.00002, 11.73]	0.22	0.26	[0.05, 1.40]	0.12
IL-6 (pg/mL)	1.03	[0.53, 2.02]	0.92	0.83	[0.16, 4.25]	0.82
IL-8 (pg/mL)	0.41	[0.06, 2.50]	0.33	0.45	[0.13, 1.59]	0.22
IL-10 (pg/mL)	-	-	-	0.002	[0.000004, 1.49]	0.06

Odds Ratio with 95% CI and logistic regression model *p*-value is shown for each study parameter and patient group. PDAC or CP vs. control: ** *p* < 0.005, * *p* < 0.05. (Logistic regression model or penalised logistic ridge regression model). PDAC (pancreatic cancer), CP (chronic pancreatitis).

**Table 3:** Characteristics of genetic instruments for serological markers.

Proxied Biomarker	Genetic Study	Study N	SNP rs	Gene	R^2^	F-Statistic
SOD activity	Lewandowski Ł et al., 2020 [26]	50	rs4880 (Val16Ala)	SOD2	0.20	20.9
GPX activity	Ravn-Haren G et al., 2006 [27]	377	rs1050450 (Pro200Leu)	GPX1	0.02	6.8
MDA levels	Rhee EP et al., 2022 [28]	822	rs33965115/ rs80018995/rs59408048	CDH1/ ALPK2/SMIM21	-	-

**Table 4:** MR results for a causal relation of the biomarkers on pancreatic diseases in two independent European cohorts.

Proxied- Biomarker	Outcome	GWAS Cohort	OR	95% CI	*p*-Value
SOD activity	PDAC	FinnGen	0.97	[0.82, 1.15]	0.71
		UK Biobank	1.17	[0.98, 1.40]	0.09
	CP	FinnGen	0.98	[0.91, 1.06]	0.70
		UK Biobank	1.18	[0.92, 1.53]	0.17
GPX activity	PDAC	FinnGen	1.26	[0.76, 2.09]	0.36
		UK Biobank	1.57	[0.87, 2.82]	0.13
	CP	FinnGen	1.07	[0.85, 1.36]	0.54
		UK Biobank	0.89	[0.40, 1.98]	0.77
MDA levels	PDAC	FinnGen	1.01	[0.64, 1.58]	0.97
		UK Biobank	1.48	[0.87, 2.51]	0.15
	CP	FinnGen	0.92	[0.76, 1.12]	0.45
		UK Biobank	0.59	[0.29, 1.23]	0.16

Odds ratio, 95% CI and its correspondent *p*-value is shown for MR analyses of each proxied biomarker and PDAC or CP. Wald ratio test was used for SOD and GPX activity, and inverse-variance weighted (IVW) method was used for MDA levels. PDAC (pancreatic cancer), CP (chronic pancreatitis).

## Data Availability

The data presented in this study are available on request from the corresponding author due to ethical reasons.
